# Metabolic disturbances and worsening of atherosclerotic lesions in ApoE^-/-^ mice after cola beverages drinking

**DOI:** 10.1186/1475-2840-12-57

**Published:** 2013-04-01

**Authors:** Matilde E Otero-Losada, Santiago Mc Loughlin, Gastón Rodríguez-Granillo, Angélica Müller, Graciela Ottaviano, Marisa Moriondo, Juan C Cutrin, José Milei

**Affiliations:** 1 Instituto de Investigaciones Cardiológicas “Prof. Dr. Alberto C. Taquini” (ININCA). Facultad de Medicina, Universidad de Buenos Aires (UBA) – Consejo Nacional de Investigaciones Científicas y Técnicas (CONICET), Buenos Aires, Argentina; 2 Department of Molecular Biotechnology and Health Sciences, University of Torino, Torino, Italy

**Keywords:** Atherosclerosis, Soft drinks, Glycemia, Urea, Creatinine, Plaque area, Apo-E deficiency

## Abstract

**Background:**

Atherosclerosis is a major health burden. Metabolic disorders had been associated with large consumption of soft drinks. The rising incidence of atherosclerosis and metabolic alterations warrants the study of long-term soft drink consumption’ effects on metabolism and atherosclerosis in genetic deficiency of apolipoprotein E which typically develops spontaneous atherosclerosis and metabolic alterations.

**Methods:**

ApoE^-/-^ mice were randomized in 3 groups accordingly with free access to: water (W), regular cola (C) or light cola (L). After 8 weeks, 50% of the animals in each group were euthanized (Treatment: W_8_, C_8_, L_8_). The remaining mice (all groups) drank water for 8 weeks and were euthanized (Washout: W_16_, C_16_, L_16_). Body weight and food and drink consumption were periodically measured. Blood was collected (biochemistry). At autopsy, transverse aortic sinus sections were serially cut and stained (histomorphometry); livers and kidneys were processed (microscopy). MANOVA (identification of variance factors) was followed by ANOVA and LSD tests (within-factor differences between levels). Conventionally a p< 0.05 was considered significant.

**Results:**

Treatment increased drinking volumes (vs W_8_: 4 fold C_8_, p<0.0001; +47% L_8_, p<0.02). Only C reduced eating amounts (–54%, p<0.05 vs W_8_). I). Compared with W_8_: C_8_ developed hyperglycemia (+43%, p<0.03) and increased non-HDL cholesterol (+54%, p<0.05); L_8_ showed decreased glycemia (–15%, p<0.05 vs W_8_) and increased creatinine (2.5 fold, p<0.04), urea (+74, p<0.03) and aspartate-aminotransferase (2.8 fold, p<0.05). Hypercreatininemia was observed in L_16_ (2.7 fold vs W_16_, p<0.05). Hypertriglyceridemia (+91%, p<0.008) and hyperuremia (+68%, p<0.03) developed over time of study (age). II). Treatment caused plaque area increase (vs W_8_: 28% C_8_, p<0.02 and 50% L_8_, p<0.01; vs W_16_: 43% C_16_, p<0.05 and 68% L_16_, p<0.02) and stenosis (vs W_8_: 38% C_8_, p<0.04 and 57% L_8_, p<0.01; vs W_16_: 71% C_16_, p<0.01 and 46% L_16_, p<0.04). Age also caused plaque area increase (56%, p<0.04). Treatment- and age-effects on plaque enlargement were additive.

**Conclusion:**

Cola beverages caused atherosclerotic lesions’ enlargement with metabolic (C) or non metabolic disturbances (L). ApoE^-/-^ mice were particularly sensitive to L treatment. These findings may likely relate to caramel colorant and non-nutritive sweeteners in cola drinks and have potential implications in particularly sensitive individuals.

## Background

Atherosclerosis is a major health burden in modern society and a leading cause of death worldwide [1]. The rising consumption of soft drinks has been linked to the growing incidence of metabolic syndrome [2,3]. Previously we reported metabolic and cardiovascular alterations associated with cola drinking in normal (eugenic) rats [4,5]. The main components of metabolic syndrome (obesity, diabetes, hypertension) are known risk factors for atherosclerosis. However we have not found information concerning with the possible consequences of long-term cola drinking on atherosclerosis. The incidence of atherosclerosis and metabolic alterations is rising and warranted the study of the effects of long-term soft drink consumption on metabolism and atherosclerosis in genetic deficiency of apolipoprotein E. Apolipoprotein E deficient (ApoE^-/-^) mice are a murine model of spontaneous atherosclerosis and develops metabolic abnormalities [6].

This is a descriptive study reporting the effects of long-term consumption of regular cola (sucrose sweetened) and light cola (aspartame-acesulfame K sweetened) on a). blood chemistry composition and b). atherosclerotic lesions in the aortic sinus (morphology,morphometry and instability features). The ApoE^-/-^ mouse was used as a model of atherosclerosis and metabolic disorder because of the potential implications of the resulting information on some individuals with personal or familiar history of atherosclerosis or metabolic disorders.

## Materials and methods

### Animals

Forty-eight Apo E knockout (ApoE^-/-^) mice (25 female, 23 male) on a C57BL/6 background were obtained from the Jackson Laboratory (Bar Harbor, Maine). All animals were fed on a standard rodent commercial chow (16%–18% protein, 0.2 g% sodium, Cooperación, Buenos Aires, Argentina) *ad libitum* and housed inside an indoor laboratory facility with a 12 h light/dark cycle. The experiments were approved by The Animal Care Committee of the University of Buenos Aires and were performed in compliance with the ARRIVE guidelines on animal research [7].

### Experimental design

ApoE^-/-^ mice (8 week-old) were randomly distributed in 3 groups accordingly with free access to one of the following drinks: water (W), regular cola (C) (sucrose sweetened carbonated drink, Coca-Cola™, Argentina), or light cola (L) (low calorie aspartame–acesulfame K sweetened carbonated drink, Coca-Cola Light™, Argentina). Cola drinks had carbon dioxide content largely removed by vigorous shaking using a stirring plate and placing a magnetic bar in a container filled with the liquid prior to being offered to the animals at room temperature. After 8 weeks, 50% of the animals in each group were euthanized (Treatment: W_8_, C_8_, L_8_). The remaining mice (all groups) drank water for another 8 weeks (i.e.: 16 weeks from beginning of the study) and were then euthanized (Washout: W_16_, C_16_, L_16_). The ratio male/female was 1/1 in all groups except for L_16_ (3/5). The animals were weighed weekly. Food and drink consumption were measured twice a week. Heparinized blood was collected and plasma was separated for biochemical assays. At autopsy, transverse aortic sinus sections were serially cut and stained for histomorphometry and livers and kidneys were processed for microscopic assessment.

According to company specifications, Coca Cola™ is a carbonated water solution containing (in 100 mL): carbohydrate 10.6 g, sodium 7 mg, caffeine 11.5 mg, caramel, phosphoric acid, citric acid, vanilla extract, natural flavourings (orange, lemon, nutmeg, cinnamon, coriander, etc.), lime juice and fluid extract of coca (*Erythroxylon novogranatense*). As far as nutritional information is concerned, the only difference between regular (43 Kcal/100 mL) and light cola (0 kcal) is the replacement of carbohydrates with non-nutritive sweeteners (aspartame 24 mg/100 mL–acesulfame K 16 mg/100 mL).

### Sample handling

Animals were sacrificed under anesthesia with sodium pentobarbital and sodium diphenylhydantoin (Euthanyl®). Blood samples were obtained by ventricular puncture and plasma was separated. Commercially available kits were used for plasma analysis and measurement of: glucose using an enzymatic-colorimetric kit (Sigma Chemical Co., St. Louis, MO, kit #315–100) followed by spectrophometry; cholesterol using enzymatic kits (Sigma Chemical Co., St. Louis, MO, kit #402–20) and triglycerides (Sigma Chemical Co., St. Louis, MO, kit #344–20). Lipoproteins were separated by sequential density ultracentrifugation (density ranges: HDL 1.063–1.210 g/mL, HDL < 1.063 g/mL using a TLA-100 rotor (Beckman Instruments, Palo Alto, CA). Liver fragments, sagital sections from both kidneys, the heart and the ascending aorta were dissected and immersed in 10% buffered formaldehyde (Formalin 10% buffered solution, pH= 7.0) at room temperature for at least a 24 h fixation. After dehydration (graded ethanol series of 50%, 70%, and 100%), tissues were embedded in paraffin blocks. Six serial transverse sections (5 μm) were cut through the aorta at the origins of the aortic valve leaflets throughout the entire aortic sinus and stained with hematoxylin-eosin, Heidenhain trichrome (Azan) and orcein for elastic fiber identification.

### Morphological and morphometric study of atheroma lesions

Features of plaque instability [8] were evaluated according with the presence of 3 or more of the following criteria: thickness of the fibrous cap (thin fibrous cap was defined as 3 or fewer cell layers), size of the necrotic core (a large necrotic core was defined as occupying more than 1/3 of the volume of the plaque), intraplaque hemorrhage (defined as the presence of red blood cells independent of microvessels) and the deposition of cholesterol crystals. Results were computed as binary outcomes and the frequency for each group was determined. Each of six serial cross sections was analysed using a software-coupled (Image Pro Plus for Windows, v3) Nikon Eclipse E400 microscope and data were averaged. In addition, plaque area, intimal layer and the media layer length were measured and stenosis percentage was calculated.

### Statistical analysis

Data were overall analyzed by multiple analysis of variance test (MANOVA) to identify sources of variation. Main effects and interactions were opened, followed by one way ANOVA and post hoc tests (LSD, least significant difference) to detect differences between levels (experimental groups) within each source of variation (within factor). Conventionally a p< 0.05 was considered significant (SPSS™ version 17.0 software).

## Results

### Blood chemistry

**Figure 1:**
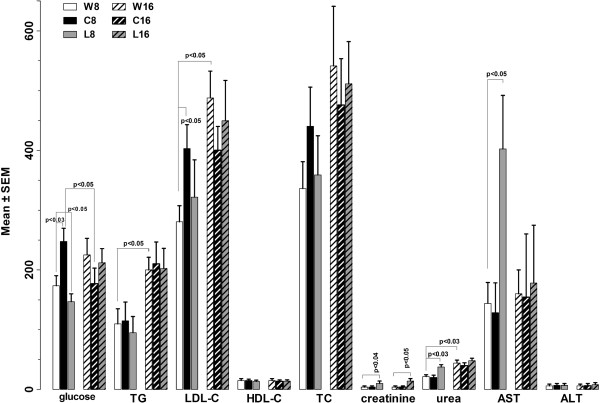
**Blood chemistry in ApoE**^**-/- **^**mice after cola beverages treatment and after washout.** Data are expressed in mg/100 mL except for enzymes (AST and ASL) activity which is expressed in units/L. An enzyme unit is defined as the amount of enzyme catalyzing the transamination of 1 μmol substrate, aspartate or alanine respectively, in 1 min at 20–25°C/68–77°F.

Cola beverages treatment increased drinking volumes (C_8_: 4 fold, p<0.0001 and L_8_: +47%, p<0.02 vs W_8_). Only regular cola reduced food consumption (–54% C_8_, p<0.05 vs W_8_) (Table 1). Compared with W_8_: C_8_ developed hyperglycemia (+43%, p<0.03) and higher non-HDL cholesterol levels (+54%, p<0.05) so that hyperglycemia accounted for 83% of non-HDL cholesterol increase (p< 0.001); L_8_ had lower glycemia (–15%, <0.05) and increased levels of creatinine (2.5 fold, p<0.04), urea (+74, p<0.03) andaspartate aminotransferase (AST) (2.8 fold, p<0.05). These changes reversed after treatment discontinuation except for persistent hypercreatininemia as found in L_16_ (2.7 fold vs W_16_, p<0.05). Over time of study (mice age effect) hypertriglyceridemia (+91%, F_1,46_=7.69, p<0.008) and hyperuremia (+68%, F_1,46_=5.01, p<0.03) developed (Figure 1).

**Table 1:** Body weight and nutritional data of ApoE-/- mice after cola beverages treatment and after washout

**Group**	**W**_**8**_	**C**_**8**_	**L**_**8**_	**W**_**16**_	**C**_**16**_	**L**_**16**_
**BW (g)**	21.4 ± 2.2	23.7 ± 3.0	20.6 ± 1.9	23.2 ± 2.0	24.5 ± 3.1	22.9 ± 2.8
**Solid intake (g)**	3.7 ± 0.3	1.7 ± 0.2	3.8 ± 0.3	3.8 ± 0.4	3.3 ± 0.3	4.1 ± 0.4
**Liquid intake (mL)**	4.9 ± 1.1	21.1 ± 1.6	7.2 ± 0.7	4.7 ± 0.4	4.5 ± 0.4	5.9 ± 0.9
**Solid intake/BW (g/g)**	0.17±0.02	0.07±0.01	0.18±0.02	0.16 ± 0.02	0.18±0.02	0.18±0.02
**Liquid intake/BW (mL/g)**	0.23±0.02	0.89±0.06	0.35±0.03	0.20 ± 0.03	0.18±0.02	0.26±0.03

### Morphological and morphometric study of atheroma lesions

**Figure 2:**
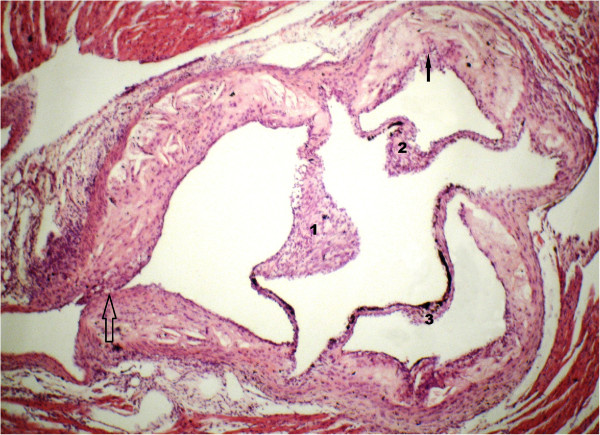
**Aortic plaque histology from an ApoE**^**-/- **^**mouse after cola beverage treatment.** The image corresponds to one of six serial sections obtained where the aortic sinus becomes the ascending aorta. The aortic plaque extends from the lumen to the internal elastic membrane. Along with this, luminal caliber is reduced with thickening or thinning and even disruption (solid arrow) of the fibrous cap. Extensive atrophy of the aortic media is observed, being replaced with plaque components, consisting of huge acellular necrotic xanthomas shaped into fibro-fatty nodules of cholesterol crystals and dense or loose connective tissue. Internal and external elastic membranes are disrupted and discontinued at the site of plaque expansion. The arrow identifies the ostia to a coronary artery which is largely obstructed by the plaque (empty arrow). Aortic leaflets are thickened and their free borders show linear calcifications and loose fibrotic excrescences (1–3). Hematoxylin and eosin x 200.

All 3 groups of mice developed qualitatively similar atherosclerotic lesions, but showed quantitative differences according with the group. The overall picture was represented by larger lesions in cola beverages treated mice (see differences below). As shown in Figure 2, the aortic plaques extended from the endothelium to the internal elastic membrane, bulging into the arterial lumen. Along with this, luminal caliber was reduced with thickening or thinning and even disruption of the fibrous cap. Extensive atrophy of the aortic media was observed, being replaced with plaque components, consisting of huge acellular necrotic xanthomas shaped into fibro-fatty nodules of cholesterol crystals surrounded by dense or loose connective tissue. Internal and external elastic membranes were disrupted and discontinued or even disappeared at the site of plaque expansion. In some sections, the ostia to the coronary arteries were mildly or largely obstructed by the plaque. Aortic leaflets were thickened and their free borders often presented linear calcifications and/or loose fibrotic excrescences (Figure 2).

**Figure 3:**
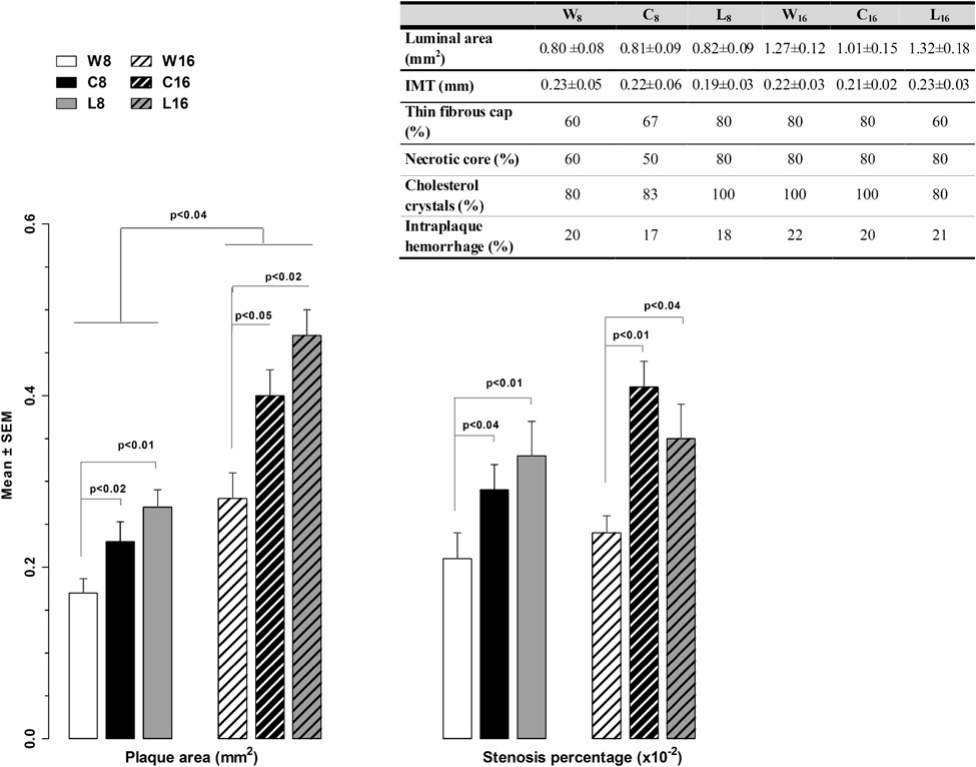
**Atherosclerosis after cola beverages treatment and after washout.** Figure depicts plaque area and stenosis percentage results. Inset table shows data for plaque instability features.

Treatment caused irreversible increase in plaque area (Figure 3) (vs W_8_: 28%, p<0.02 in C_8_ and 50%, p<0.01 in L_8_; vs W_16_: 43%, p<0.05 in C_16_ and 68%, p<0.02 in L_16_) and stenosis (vs W_8_: 38%, p<0.04 in C_8_ and 57%, p<0.01 in L_8_; vs W_16_: 71%, p<0.01 in C_16_ and 46%, p<0.04 in L_16_). Age was overall associated with plaque area increase (56%, F_2,42_=3.48, p<0.04). Treatment and age had additive effects on plaque area and did not interact with each other (F_Treatment*Age 2,42_=2.17, N.S.).

Plaque instability features showed no variation related to group (inset table in Figure 3).

### Liver and kidney morphology

Treatment with cola beverages had no effect on the morphology or the aging process of these organs. Accordingly all mice regardless of the group showed a mild degree of liver steatosis and scattered foci of acinar inflammation, whereas only a slight chronic interstitial inflammatory infiltration at the level of the transition between the outer and internal medullar zones were observed in the kidneys (not shown).

## Discussion

In this paper, whereas predictable results were observed after the administration of regular cola to ApoE^-/-^ mice, light cola consumption revealed unexpected observations. Neither regular cola nor light cola drinking modified body weight. Interestingly, solid food intake decreased in the regular cola group, likely as the result of drinking large volumes of regular cola which provided excess caloric intake. We reported the same nutritional behavior in eugenic rats after drinking regular cola for a long period [4,5].

### Blood chemistry

Regular cola drinking (C, sucrose sweetened) resulted in hyperglycemia which largely accounted for the observed increase in the non-HDL cholesterol fraction. These changes reversed after the washout period. Differently, light cola drinking (L, aspartame–acesulfame K sweetened) induced a mild decrease in glycemia, with hypercreatininemia, hyperuremia and increase in AST, all of which reversed after washout except for hypercreatininemia. Acesulfame K and other non-nutritive sweeteners (but not aspartame) have been reported to activate enteroendocrine sweet taste receptors and release incretins which stimulate pancreatic insulin secretion [9-11]. Reasonably, this mechanism might help to understand the decrease in glycemia observed in the L group. Likewise, phenylalanine, 40% of metabolilzed aspartame [12], acting synergically with solid food and stimulating insulin release [13] might participate in the decrease in glycemia in L group.

Reduced creatinine clearance has been reported in 8 week-old (young) ApoE^-/-^ mice [14] indicating some degree of vulnerability in glomerular filtration. Oxidative stress is associated with either high uremia levels or methanol ocurrence in blood [15]. In L mice uremic toxicity and increased oxidative stress may correlate with a pro-inflammatory condition. However liver pathology was not detectable by optic microscopy in this study. A temporal delay between biochemical changes and morphological alterations may partly contribute to explain the lack of correlation.

Over time of study hypertriglyceridemia and hyperuremia developed in all groups irrespective of drink treatment likely as a result of the aging process which is typically accelerated in ApoE^-/-^ mice.

### Arterial pathology

Cola drinking resulted in enlargement of atherosclerotic lesions and increased stenosis. Time over study (aging) was *per se* associated with both increased plaque area and stenosis. After washout further worsening of atherosclerotic lesions (i.e. enlargement of plaque area and increase in stenosis degree) observed in mice that had consumed colas was interpreted as a result of aging.

Recently, safrole-2^′^,3^′^-oxide (main component of sassafras oil in nutmeg) which is found in cola beverages, has been reported to aggravate atherosclerosis in ApoE^−/−^ mice [16].

It is known that lipid profile and atherosclerosis are influenced by gender in mice, particularly in ApoE^−/−^ mice [17-19]. However, the influence of gender on atherosclerosis and the protective effect of estrogens are not yet clear [20-22]. We observed only a trend towards increased total cholesterol levels in males compared to females. One likely explanation is that the number of animals per gender was too small.

### Possible mechanisms responsible for the effects of cola drinking on aortic sinus plaque

Atherosclerosis in ApoE^-/-^ mice can be affected by several factors. Defective insulin secretion, smaller islet mass and islet inflammation have been found in atherosclerosis-susceptible B6.ApoE^-/-^ mice compared to atherosclerosis-resistant BALB.ApoE^-/-^ mice (C57BL/6 and BALB/cJ respectively) [23]. Present finding of hyperglycemia after chronic regular cola drinking may be consistent with such possibility, i.e: reduced beta cell mass and insulin secretion which have not been evaluated in this study. We have observed islet mass reduction with hyperglycemia after chronic regular cola drinking in rats (unpublished observations). Genetic deficit or pharmacological blockade of angiotensin receptor 1 (AT_1_R) attenuates atherosclerosis and improves endothelial function in experimentally induced diabetes in ApoE^−/−^ mice via peroxisome proli-ferator-activated receptor γ (PPARγ) pathway [24,25]. We have found no reports relating cola drinking with ATR1 or the PPARγ pathway.

Caramel colorant, contained in both regular and light cola beverages. is a source of advanced glycation end products (AGEs) [3,4] promoting a proatherogenic pro-oxidative status [26].

Uremia and even mild renal dysfunction have been reported to cause a dramatic increase in plaque size and aggressive morphology (foam cell rich soft plaques) in ApoE^-/-^ mice [27]. High uremia, which has been associated with a prooxidative and proinflamatory condition, was presently observed after chronic light cola drinking [15]. In addition, the generation of ROS by methanol originated by aspartame cleavage offers a reasonable mechanism underlying the effects of light cola drinking. Also unmetabolized aspartame, 10–15% of ingested aspartame [28], modifies the intestinal environment and triggers inflammatory (pro-atherogenic) processes [11].

In this study light cola drinking caused hyperuremia and atherosclerosis in 16 weeks-old mice (8 weeks-old mice at the beginning of the study + 8 week-treatment) while these conditions typically develop in aged ApoE-/- mice [29-31]. Accordingly, it follows that long term L consumption might precipitate aging mechanisms in arterial vessels in susceptible hosts.

## Conclusions

ApoE^-/-^ mice were particularly sensitive to the effects of light cola drinking. Increase in AST, uremia and creatininemia suggest functional interference at one or more levels (liver, kidney, muscle) and warrants future research.

## Abbreviations

W: Water; C: Regular cola (sucrose sweetened); L: Light cola (aspartame-acesulfame K sweetened); ApoE-/-: Apolipoprotein E deficient mice; HDL: High density lipoprotein; AST: Aspartate-aminotransferase; AGE: Advanced glycation end products.

## Competing interests

The authors declare that they have no competing interests.

## Authors’ contributions

MJ conceived and designed the study. RGG provided the transgenic mice strain. MJ and McLS performed morphological and morphometric evaluation of aortic lesions. CJC performed pathological evaluations of liver and kidney. MA and OG assisted in mice treatment. They and MM processed tissues for morphological and pathological exams. O-LME performed statistical analysis and plotting of results. O-LME and MJ wrote the paper. All authors contributed to the final discussion, read and approved the final manuscript.

## References

[B1] Vasquez EC Peotta VA Gava AL Pereira TM Meyrelles SS Cardiac and vascular phenotypes in the apolipoprotein E-deficient mouse J Biomed Sci 2012 19 22 10.1186/1423-0127-19-22 22330242 PMC3306747

[B2] Dhingra R Sullivan L Jacques PF Wang TJ Fox CS Meigs JB D’Agostino RB Gaziano JM Vasan RS Soft drink consumption and risk of developing cardiometabolic risk factors and the metabolic syndrome in middle-aged adults in the community Circulation 2007 116 480 488 10.1161/CIRCULATIONAHA.107.689935 17646581

[B3] Nseir W Nassar F Assy N Soft drinks consumption and nonalcoholic fattyliver disease World J Gastroenterol 2010 16 2579 2588 10.3748/wjg.v16.i21.2579 20518077 PMC2880768

[B4] Milei J Otero Losada M Gómez Llambí H Grana DR Suárez D Azzato F Ambrosio G Chronic cola drinking induces metabolic and cardiac alterations in rats World J Cardiol 2011 3 111 116 10.4330/wjc.v3.i4.111 21526048 PMC3082734

[B5] Otero-Losada ME Grana DR Müller A Ottaviano G Ambrosio G Milei J Lipid profile and plasma antioxidant status in sweet carbonated beverage-induced metabolic syndrome in rat Int J Cardiol 2011 146 106 109 10.1016/j.ijcard.2010.09.066 21055834

[B6] Zhang SH Reddick RL Piedrahita JA Maeda N Spontaneous hypercholesterolemia and arterial lesions in mice lacking apolipoprotein E Science 1992 258 468 471 10.1126/science.1411543 1411543

[B7] Kilkenny C Browne WJ Cuthill IC Emerson M Altman DG Improving bioscience research reporting: the ARRIVE guidelines for reporting animal research PLoS Biol 2010 8 e1000412 10.1371/journal.pbio.1000412 20613859 PMC2893951

[B8] Bea F Blessing E Bennett BJ Kuo CC Campbell LA Kreuzer J Rosenfeld ME Chronic inhibition of cyclooxygenase-2 does not alter plaque composition in a mouse model of advanced unstable atherosclerosis Cardiovasc Res 2003 60 198 20 10.1016/S0008-6363(03)00464-4 14522423

[B9] Brown RJ Walter M Rother KI Ingestion of diet soda before a glucose load augments glucagon-like peptide-1 secretion Diabetes Care 2009 32 2184 2186 10.2337/dc09-1185 19808921 PMC2782974

[B10] Brown RJ Rother KI Non-nutritive sweeteners and their role in the gastrointestinal tract J Clin Endocrinol Metab 2012 97 2597 2605 10.1210/jc.2012-1475 22679063 PMC3410280

[B11] Pepino MY Bourne C Non-nutritive sweeteners, energy balance, and glucose homeostasis Curr Opin Clin Nutr Metab Care 2011 14 391 395 10.1097/MCO.0b013e3283468e7e 21505330 PMC3319034

[B12] Humphries P Pretorius E Naudé H Direct and indirect cellular effects of aspartame on the brain Eur J Clin Nutr 2008 62 451 462 10.1038/sj.ejcn.1602866 17684524

[B13] Calbet JA MacLean DA Plasma glucagon and insulin responses depend on the rate of appearance of amino acids after ingestion of different protein solutions in humans J Nutr 2002 132 2174 2182 12163658 10.1093/jn/132.8.2174

[B14] Balarini CM Oliveira MZ Pereira TM Silva NF Vasquez EC Meyrelles SS Gava AL Hypercholesterolemia promotes early renal dysfunction in apolipoprotein E-deficient mice Lipids Health Dis 2011 10 220 10.1186/1476-511X-10-220 22117541 PMC3247872

[B15] D’Apolito M Du X Zong H Catucci A Maiuri L Trivisano T Pettoello-Mantovani M Campanozzi A Raia V Pessin JE Brownlee M Giardino I Urea-induced ROS generation causes insulin resistance in mice with chronic renal failure J Clin Invest 2010 120 203 213 10.1172/JCI37672 19955654 PMC2798674

[B16] Su L Zhang H Zhao J Zhang S Zhang Y Zhao B Miao J Safrole-2^′^,3^′^-oxide induces atherosclerotic plaque vulnerability in apolipoprotein E-knockout mice Toxicol Lett 2013 217 129 136 10.1016/j.toxlet.2012.12.011 23270965

[B17] Meyrelles SS Peotta VA Pereira TM Vasquez EC Endothelial dysfunction in the apolipoprotein E-deficient mouse: insights into the influence of diet, gender and aging Lipids Health Dis 2011 10 211 10.1186/1476-511X-10-211 22082357 PMC3247089

[B18] Pereira TM Nogueira BV Lima LC Porto ML Arruda JA Vasquez EC Meyrelles SS Cardiac and vascular changes in elderly atherosclerotic mice: the influence of gender Lipids Health Dis 2010 9 87 10.1186/1476-511X-9-87 20723257 PMC2936359

[B19] Pérez-López FR Larrad-Mur L Kallen A Chedraui P Taylor HS Gender differences in cardiovascular disease: hormonal and biochemical influences Reprod Sci 2010 17 511 531 10.1177/1933719110367829 20460551 PMC3107852

[B20] Thomas CM Smart EJ Gender as a regulator of atherosclerosis in murine models Curr Drug Targets 2007 8 1172 1180 10.2174/138945007782403874 18045095

[B21] Freudenberger T Oppermann M Heim HK Mayer P Kojda G Schrör K Fischer JW Proatherogenic effects of estradiol in a model of accelerated atherosclerosis in ovariectomized ApoE-deficient mice Basic Res Cardiol 2010 105 479 486 10.1007/s00395-010-0091-6 20177692

[B22] Caligiuri G Nicoletti A Zhou X Törnberg I Hansson GK Effects of sex and age on atherosclerosis and autoimmunity in apoE-deficient mice Atherosclerosis 1999 145 301 308 10.1016/S0021-9150(99)00081-7 10488957

[B23] Li J Wang Q Chai W Chen MH Liu Z Shi W Hyperglycemia in apolipoprotein E-deficient mouse strains with different atherosclerosis susceptibility Cardiovasc Diabetol 2011 10 117 10.1186/1475-2840-10-117 22204493 PMC3273441

[B24] Tiyerili V Becher UM Aksoy A Lütjohann D Wassmann S Nickenig G Mueller CF AT1-receptor-deficiency induced atheroprotection in diabetic mice is partially mediated via PPARgamma Cardiovasc Diabetol 2013 12 30 10.1186/1475-2840-12-30 23374104 PMC3667017

[B25] Wassmann S Czech T van Eickels M Fleming I Bohm M Nickenig G Inhibition of diet-induced atherosclerosis and endothelial dysfunction in apolipoprotein E/angiotensin II type 1A receptor double-knockout mice Circulation 2004 110 3062 3067 10.1161/01.CIR.0000137970.47771.AF 15277329

[B26] Pasqui AL Bova G Maffei S Auteri A Immune factors in atherosclerosis Ann Ital Med Int 2005 20 81 89 16052840

[B27] Buzello M Törnig J Faulhaber J Ehmke H Ritz E Amann K The apolipoprotein e knockout mouse: a model documenting accelerated atherogenesis in uremia J Am Soc Nephrol 2003 14 311 316 10.1097/01.ASN.0000045048.71975.FC 12538731

[B28] Creppy EE Baudrimont I Anne-Marie W How aspartame prevents the toxicity of ochratoxin A J Toxicol Sci 1998 23 165 172 10.2131/jts.23.SupplementII_165 9760456

[B29] Yu J Deng M Zhao J Huang L Decreased expression of klotho gene in uremic atherosclerosis in apolipoprotein E-deficient mice Biochem Biophys Res Commun 2010 391 261 266 10.1016/j.bbrc.2009.11.046 19912987

[B30] Ivanovski O Szumilak D Nguyen-Khoa T Ruellan N Phan O Lacour B Descamps-Latscha B Drüeke TB Massy ZA The antioxidant N-acetylcysteine prevents accelerated atherosclerosis in uremic apolipoprotein E knockout mice Kidney Int 2005 67 2288 2294 10.1111/j.1523-1755.2005.00332.x 15882270

[B31] Massy ZA Ivanovski O Nguyen-Khoa T Angulo J Szumilak D Mothu N Phan O Daudon M Lacour B Drüeke TB Muntzel MS Uremia accelerates both atherosclerosis and arterial calcification in apolipoprotein E knockout mice J Am Soc Nephrol 2005 16 109 116 15563564 10.1681/ASN.2004060495

